# Tumor cell death after electrotransfer of plasmid DNA is associated with cytosolic DNA sensor upregulation

**DOI:** 10.18632/oncotarget.24816

**Published:** 2018-04-10

**Authors:** Katarina Znidar, Masa Bosnjak, Nina Semenova, Olga Pakhomova, Loree Heller, Maja Cemazar

**Affiliations:** ^1^ Faculty of Health Sciences, University of Primorska, Izola, Slovenia; ^2^ Department od Experimental Oncology, Institute of Oncology Ljubljana, Ljubljana, Slovenia; ^3^ Frank Reidy Research Center of Bioelectrics, Old Dominion University, Norfolk, Virginia, USA; ^4^ School of Medical Diagnostic and Translational Sciences, Collage of Health Sciences, Old Dominion University, Norfolk, Virginia, USA

**Keywords:** plasmid DNA, pattern recognition receptors, mammary adenocarcinoma cells, fibrosarcoma cells, electroporation, Immunology

## Abstract

Cytosolic DNA sensors are a subgroup of pattern recognition receptors (PRRs) and are activated by the abnormal presence of the DNA in the cytosol. Their activation leads to the upregulation of pro-inflammatory cytokines and chemokines and can also induce cell death. The presence of cytosolic DNA sensors and inflammatory cytokines in TS/A murine mammary adenocarcinoma and WEHI 164 fibrosarcoma cells was demonstrated using real time reverse transcription polymerase chain reaction (RT-PCR), western blotting and enzyme-linked immunosorbent assay (ELISA). After electrotransfer of plasmid DNA (pDNA) using two pulse protocols, the upregulation of DNA-depended activator of interferon regulatory factor or Z-DNA binding protein 1 (DAI/ZBP1), DEAD (Asp-Glu-Ala-Asp) box polypeptide 60 (DDX60) and interferon-inducible protein 204 (p204) mRNAs was observed in both tumor cell lines, but their expression was pulse protocol dependent. A decrease in cell survival was also observed; it was cell type, DNA concentration and pulse protocol dependent. Furthermore, the different protocols of electrotransfer led to different cell death outcomes, necrosis and apoptosis, as indicated by an annexin V and 7AAD assays. The obtained data provide new insights on the presence of cytosolic DNA sensors in tumor cells and the activation of different types of cells death after electrotransfer of pDNA. These observations have important implications on the planning of gene therapy or DNA vaccination protocols.

## INTRODUCTION

Electroporation (EP) is a delivery method in which cells are exposed to electric pulses with specific intensities and durations in order to increase the permeability of the cell membrane, enabling transition of polar molecules into the cells. In biomedicine, this technique is widely used to transform bacteria and transfect eukaryotic cells and tissues [1]. Furthermore, this technique has reached clinical applications. In oncology, EP is used clinically to facilitate the entry of the chemotherapeutic agents bleomycin and cisplatin into the cells of several tumor types (electrochemotherapy or ECT), resulting in increased antitumor effectiveness of chemotherapeutic drugs leading to high percentage of complete tumor regressions [2–6]. Currently, ECT is performed in more than 140 cancer centers in Europe and is included in the national guidelines for treatment in UK, Germany, Slovenia and other EU countries. Another application of electroporation that has reached clinical trials is gene electrotransfer, where electroporation is used for transfection of cells within tissues with plasmid DNA (pDNA), generally for the purpose of cancer therapy, cancer vaccines, or infectious disease vaccines. In oncology, several clinical trials are ongoing for several types of cancer using pDNA encoding different therapeutic molecules, either immunomodulatory molecules such as interleukin-12 [7] or tumor associated antigens [8–11].

Although EP is used clinically, the electrical parameters for gene electrotransfer differ substantially depending on the therapeutic use. Tissue type, local or systemic type of expression as well as its duration, which are crucial for successful treatment outcomes, also affect the choice of electrical parameters for effective gene transfer. Currently, no universal pulse protocol exists for specific tissue applications, although three primary pulse protocols are used for tissue transfection. In the first, the same electrical parameters are used as in electrochemotherapy [12]. Plasmid delivery with this pulse protocol has reached tumor targeted clinical trials in the United States for melanoma [7], Merkel cell carcinoma [13], squamous cell carcinoma, and triple-negative breast cancer (ClinicalTrials.gov). In the second, electric pulses of lower amplitude and longer duration are used [14, 15]. This pulse type has reached clinical trials for muscle delivery [8]. Finally, a combination of high voltage and low voltage electric pulses can be utilized [16, 17]. This combination of pulse types has reached clinical trials for intratumor delivery [18].

At the cellular level, the mechanisms of electrotransfer-mediated pDNA entry are not fully understood. After the exposure of the cell to electric pulses in the presence of pDNA, a DNA-membrane complex is formed on the membrane facing the cathode [19, 20], then this complex enters the cells via endocytosis [21] or macropinocytosis [22]. DNA escapes intracellular vesicles to enter the cell’s nucleus to be transcribed then translated into the therapeutic protein. Only DNA that enters the cells via these mechanisms is transcribed, because inhibitors of endocytosis almost completely abrogate the expression of pDNA-encoded genes [23, 24].

In our previous study, we showed that the mRNAs and proteins of the receptors of the innate immune system that recognize foreign DNA (cytosolic DNA sensors) were upregulated after vector pDNA (plasmid backbone without therapeutic gene) electrotransfer in B16F10 melanoma cells [25]. This indicated that DNA is detected in the cytosol after endosomal escape or hypothetically it may also enter directly via electropores formed in the cell membrane [26–28]. Cytosolic DNA sensors are a subgroup of pattern recognition receptors (PRRs) and are activated by the abnormal presence of the DNA in the cytosol. Their activation leads to the upregulation of pro-inflammatory cytokines and chemokines, which may induce and inflammatory immune response. This activation can also lead to cell death [29–33]. In addition to upregulation of several cytosolic DNA sensors, in our previous studies, we showed increased cell death *in vitro* and complete regression of tumors *in vivo* [34, 35]. These effects were accompanied by increased production of interferon β (IFNβ) both *in vitro* and *in vivo* implicating paracrine-autocrine signaling leading to cell death [25].

Tumor regression and increased cell death *in vitro* have been demonstrated for other tumors such as sarcomas and carcinomas, and for tumor cell lines, following electrotransfer of pDNA devoid of therapeutic genes [36–45]. However, it is not known whether other tumor cell types of mesoderm origin (fibrosarcoma) and ectoderm origin (carcinoma) respond to pDNA electrotransfer in a manner similar to melanoma cells. Because the activation of immune system is important for planning and developing new treatment modalities for cancer, three different types of DNA electrotransfer pulse protocols were evaluated for potential upregulation of cytosolic DNA sensors and the downstream consequences of their activation, such as the production of pro-inflammatory molecules and induced cell death.

## RESULTS

### Transfection efficiency, cytotoxicity and ATP levels

**Figure 1:**
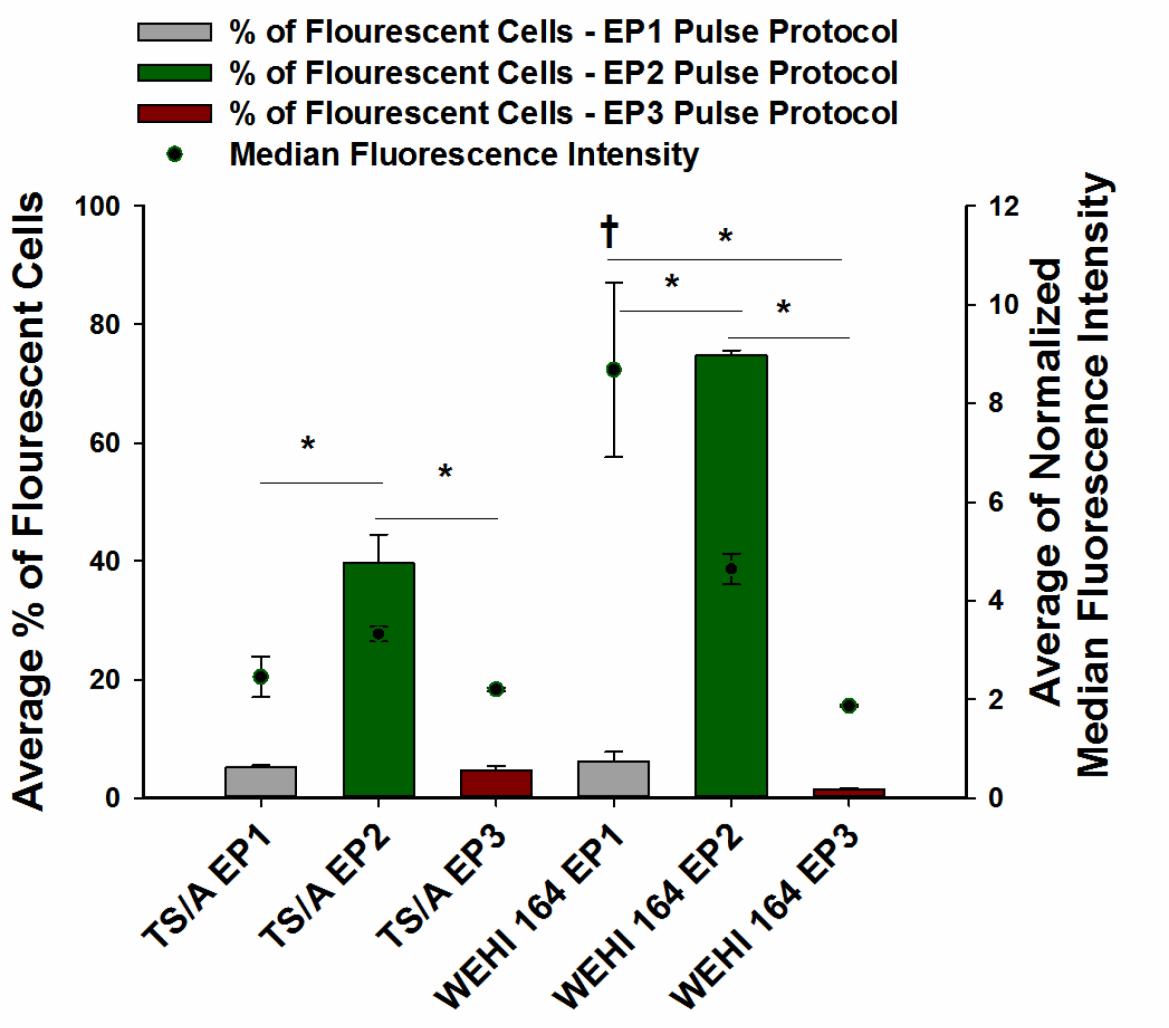
Transfection efficiency of TS/A and WEHI 164 cell lines after pEGFP-N1 electrotransfer using three different pulse protocols of DNA electrotransfer pEGFP-N1 was electrotransfered by delivery of eight 5 ms pulses with a voltage to distance ratio of 600 V/cm, frequency 1 Hz (EP1), six 100 µs pulses with a voltage to distance ratio of 1300 V/cm, frequency 4 Hz (EP2) or with combination of one 100 µs pulse with a voltage to distance ratio 600 V/cm and four 100 ms pulses with a voltage to distance ratio 80 V/cm, duration, frequency 1Hz (EP3) using plate electrode. ^*^statistically significant difference of percentage of fluorescent cells between electrotransfer protocol groups (*P* < 0.05). ^†^Statistically significant difference between the mean values of median fluorescence intensity of cells receiving the EP1 protocol and fluorescence intensity of cells receiving the EP2 and EP3 pulse protocols.

Transfection efficiency, cell survival, and ATP levels were quantified after electrotransfer into TS/A and WEHI 164 cells using three different pulse protocols. The number of transfected cells, or transfection efficiency, was pulse protocol dependent. Pulse protocol EP2 produced a significantly higher transfection efficiency in both cell lines than the other pulse protocols, with 39.7 ± 4.8% fluorescent cells in TS/A cell line and 74.9 ± 0.8% in WEHI 164. Both the EP1 and EP3 pulse protocols transfected less than 10% of cells (Figure 1).

**Figure 2:**
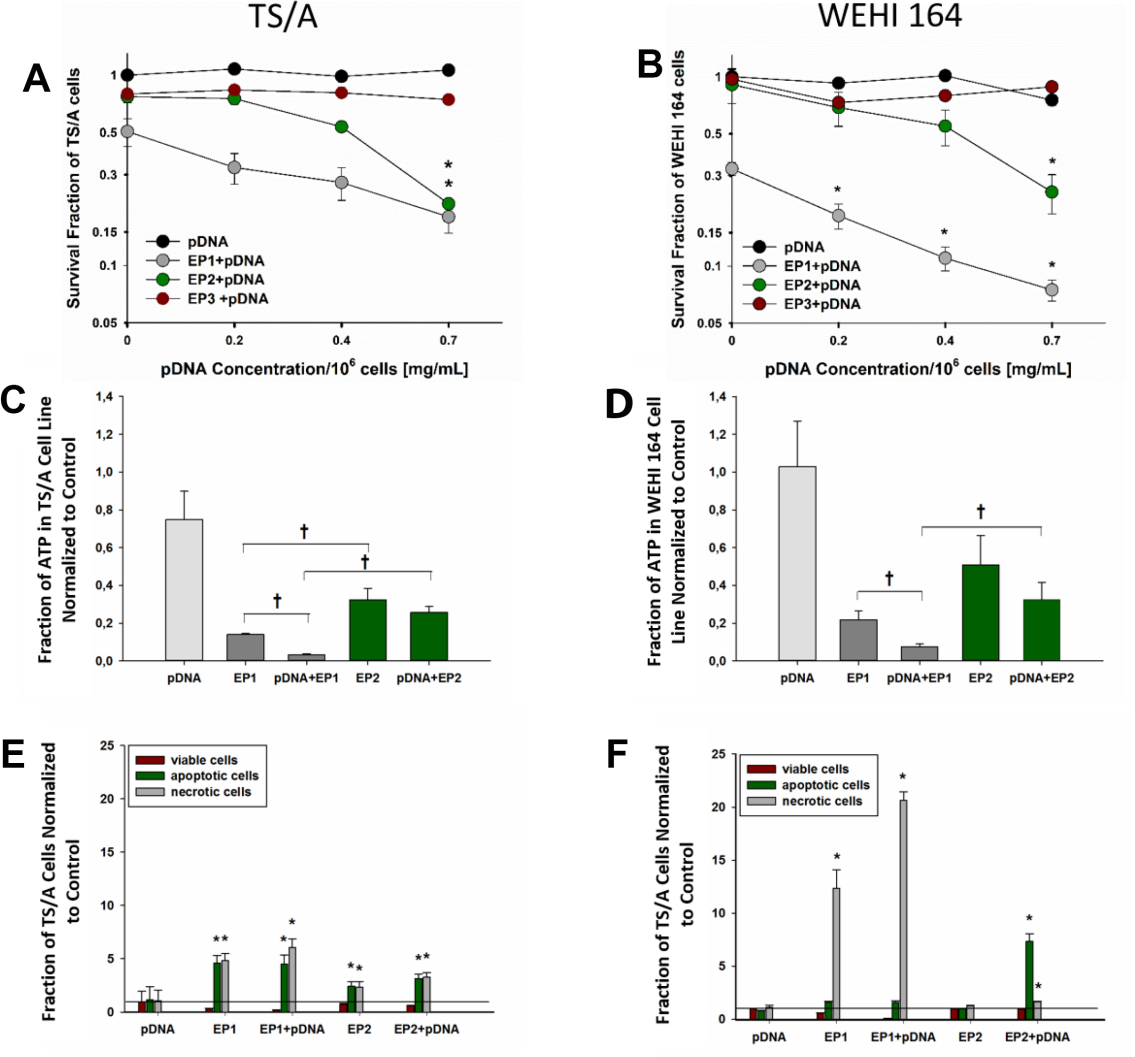
Cell survival, ATP level determination and cell death mechanism after electrotransfer in TS/A and WEHI 164 cell lines Cell survival was measured 72 hours after electrotransfer of pDNA using the pulse protocols described in methods and Figure 1 in (**A**) TS/A cells and (**B**) WEHI 164 cells. The survival fraction was normalized to an unexposed control group. The concentrations on X-axis represent final pDNA concentrations; 10 µg/10^6^ cells, 20 µg/10^6^ cells and 35 µg/10^6^ cells in 50 µl of total volume, respectively. The percentage of ATP inside (**C**) TS/A and (**D**) WEHI 164 cells was determined immediately after electrotransfer. Cell death mechanisms were quantified in (**E**) TS/A and cells (**F**) WEHI 164 cells for the EP1 and EP2 electrotransfer protocols in the presence and absence of pDNA by flow cytometry for Annexin V and 7-AAD. ^*^Statistically significant difference compared to different electrotransfer pulse protocol (EP) groups (*P* < 0.05). ^†^statistically significant difference between indicated groups (*P* < 0.05). ^#^statistically significant difference from pDNA group (*p* < 0.05).

Although the transfection efficiency varied greatly between the pulse protocols, in TS/A cells no statistically significant changes in median fluorescence intensity between pulse protocols were observed. Whereas, in WEHI 164 cells, the fluorescence intensity of cells following transfection with the EP1 pulse protocol was statistically significantly higher than fluorescence intensity of cells transfected with the other two pulse protocols, indicating that although this pulse protocol is very cytotoxic (Figure 2), it enables higher numbers of plasmid copies to enter the cell’s nucleus for expression.

The EP1 pulse protocol was less cytotoxic to TS/A cells (Figure 2A) than to WEHI 164 cells (Figure 2B); the application of EP1 pulses alone decreased the survival fraction by 49% in TS/A cells and 67% in WEHI 164 cells. Cell survival was less affected in either cell line after application of pulse protocols EP2 or EP3 alone (Figure 2A, 2B). For pulse protocols EP1 and EP2, the addition of pDNA further decreased cell viability in a dose-dependent manner that was particularly detectable in WEHI 164 cells (Figure 2B). On the other hand, the addition of pDNA had no effect on cell survival after the application of the EP3 pulse protocol; therefore, this protocol was not tested in subsequent experiments.

Thirty minutes after gWiz Blank electrotransfer with EP1 and EP2, ATP levels were measured (Figure 2C, 2D, Supplementary Table 2) to determine if the observed difference in the cytotoxic effects between electrotransfer pulse protocols was due to ATP leakage, which can cause cell necrosis.

A significant decrease in ATP levels was observed after pulse delivery. The decrease after EP1 pulse delivery alone was significantly greater than the decrease after EP2 pulses alone. The addition of pDNA further reduced ATP levels in cells using EP1 but not EP2 for delivery of pDNA (*p <* 0.05).

### Cell death mechanisms and morphology

We investigated the mechanisms of cell death using pulse protocols EP1 and EP2. Differences in the level of apoptosis (early apoptosis) or necrosis (accompanied by late apoptosis) depended on the cell line and the electrotransfer protocol. In TS/A cells, both necrotic and apoptotic cells were detected with both EP pulse protocols 20 hours after electrotransfer of pDNA (Figure 2E); cell death was higher after EP1 pulses (Figure 2A). In WEHI 164 cells, we observed a greater number of apoptotic cells after pDNA electrotransfer using pulse protocol EP2 (*p <* 0.05), and a greater number of necrotic cells after pDNA electrotransfer using pulse protocol EP1 (*p <* 0.05) (Figure 2F). These results were confirmed morphologically using Giemsa staining perfomed 6 hours after electrotranfer of pDNA (Supplementary Figures 1, 2). Several necrotic cells were observed after electrotransfer of pDNA using pulse protocol EP1 in both cell lines. Based on Giemsa staining, we confirmed that a greater quantity of apoptotic cells were produced after electrotransfer using the EP2 pulse protocol in WEHI 164 cells.

### Increased expression of DNA sensors depends on pulse protocol selection and cell type

The mRNAs for several PRRs were detected in both TS/A (Table 1) and WEHI 164 cells (Table 2), while the mRNAs for toll-like receptor 9 (TLR9), retinoic acid inducible gene I (RIG1), and absent in melanoma 2 (AIM2) were not. SRY box 2 (SOX2) mRNA was detected in WEHI 164 but not TS/A cells. After vector pDNA electrotransfer, several DNA sensors were upregulated at the mRNA level. Increased expression of DDX60, DAI/ZBP1 and p204 mRNAs occurred in both cell lines with variation in the level of expression depending on the cell line and the EP pulse protocol used (Tables 1, 2). There were, however, differences between cell lines. DEAH (Asp-Glu-Ala-His) box helicase 36 (DHX36) was significantly upregulated in TS/A cells after pDNA delivery with EP1. The mRNAs for leucine-rich repeat flightless-interacting protein 1 (LRRFIP1), interferon activated gene 202 (p202), and p204 were minimally but significantly upregulated after pDNA delivery with EP2 in WEHI 164 cells.

**Table 1:** Fold changes in mRNA levels of endosomal and cytosolic DNA sensors in TS/A tumor cells 4 hours after pDNA electrotransfer

TS/A		control		gWiz Blank		EP1		EP2		EP3		pDNA+EP1		pDNA+EP2		pDNA+EP3
	*n*	foldex ± SE	*n*	foldex ± SE	*n*	foldex ± SE	*n*	foldex ± SE	*n*	foldex ± SE	*n*	foldex ± SE	*n*	foldex ± SE	*n*	foldex ± SE
TLR9	8	/	6	/	3	/	2	/	3	/	4	/	3	/	3	/
RIG-1	8	/	6	/	3	/	2	/	3	/	4	/	3	/	3	/
**DDX60**	9	1.2 ± 0.3	6	0.7 ± 0.3	4	0.7 ± 0.2	2	1.7 ± 1.2	3	0.8 ± 0.0	**7**	3.2 ± 0.7^*^	**5**	4.0 ± 0.9^*^	3	1.0 ± 0.2
DHX9	8	1.2 ± 0.2	6	0.3 ± 0.1	4	0.4 ± 0.1	2	1.7 ± 1.2	3	1.3 ± 0.2	4	0.1 ± 0.0	3	1.4 ± 0.3	3	0.8 ± 0.1
**DHX36**	9	1.3± 0.4	6	0.6± 0.1	4	0.9 ± 0.3	2	1.7 ± 1.2	3	0.9 ± 0.1	**4**	7.7 ± 1.2^*^	5	1.9 ± 1.0	3	0.6 ± 0.1
AIM	8	/	5	/	3	/	2	/	3	/	3	/	3	/	3	/
cGAS	7	1.4 ± 0.5	5	1.1 ± 0.3	3	1.0 ± 0.4	2	1.9 ± 1.1	3	1.7 ± 0.1	3	1.6 ± 0.6	3	1.0 ± 0.1	3	1.1 ± 0.1
**DAI/ZBP1**	8	1.7 ± 0.5	4	1.5 ± 1.2	3	0.7 ± 0.1	2	1.7 ± 1.2	3	1.1 ± 0.2	**5**	14.1 ± 3.9^*^	**3**	3.0 ± 0.6^*^	3	1.0 ±0.1
DDX41	6	1.1 ± 0.2	4	1.3 ± 0.2	3	1.6 ± 0.4	2	1.8 ± 1.1	3	1.2 ± 0.2	3	1.3 ± 0.5	3	1.3 ± 0.2	3	0.9 ± 0.0
LRRFIP1	6	0.8 ± 0.1	5	0.8 ± 0.1	3	1.1 ± 0.2	2	1.8 ± 1.1	3	1.2 ± 0.1	3	2.2 ± 0.8	3	2.8 ± 1.2	3	0.9 ± 0.0
P202	7	1.2 ± 0.2	5	0.8 ± 0.3	3	0.7 ± 0.3	2	1.7 ± 1.2	3	0.9 ± 0.0	3	2.8 ± 1.0	3	4.1 ± 2.4	3	1.0 ± 0.1
**P204**	3	1.0 ± 0.2	2	1.0 ± 0.1	2	0.6 ± 0.1	2	1.9 ± 0.5	3	1.2 ± 0.1	3	1.1 ± 0.1	**3**	3.4 ± 0.3^*^	**3**	2.1 ± 0.2^*^
SOX2	4	/	2	/		/	2	/	2	/	3	/	3	/	2	/
MRE1	4	1.2 ± 0.2	2	1.5 ± 0.4	2	0.8 ± 0.1	2	1.0 ± 0.1	2	0.6 ± 0.0	3	0.8 ± 0.2	3	0.9 ± 0.1	2	0.5 ± 0.0
Ku70	4	0.9 ± 0.2	2	1.0 ± 0.1	2	1.1 ± 0.0	2	0.8 ± 0.1	2	1.0 ± 0.0	3	1.0 ± 0.1	3	0.8 ± 0.1	2	1.0 ± 0.0

Legend: cGAS, cyclic guanosine monophosphate-adenosine monophosphate synthase; DAI/ZBP1, DNA-dependent activator of interferon regulatory factor; DDX41, DEAD (Asp-Glu-Ala-Asp) box polypeptide 41; DDX60, DEAD (Asp-Glu-Ala-Asp) box polypeptide 60; DHX9, DEAH (Asp-Glu-Ala-His)box helicase 9; DHX36, DEAH (Asp-Glu-Ala-His) box helicase 36; LRRFIP1, leucine-rich repeat flightless-interacting protein 1; p204, interferon activated gene 204; p202, interferon activated gene 202; MRE11, meiotic recombination 11 homolog, double strand break repair nuclease; SOX2, SRY (sex determining region Y)-box 2; Ku70, Lupus Ku autoantigen protein p70^*^statistically significant difference compared to control group (*P* < 0.05); ND, not detected.

**Table 2:** Fold changes in mRNA levels of endosomal and cytosolic DNA sensors in WEHI 164 tumor cells 4 hours after pDNA electrotransfer

WEHI 164		control		gWiz Blank		EP 1		EP 2		EP 3		pDNA+EP1		pDNA+EP2		pDNA+EP3
	*n*	foldex ± SE	*n*	foldex ± SE	*n*	foldex ± SE	*n*	foldex ± SE	*n*	foldex ± SE	*n*	foldex ± SE	*n*	foldex ± SE	*n*	foldex ± SE
TLR9	4	/	3	/	3	/	3	/	3	/	5	/	5	/	3	/
RIG-1	4	/	3	/	3	/	3	/	3	/	5	/	5	/	3	/
**DDX60**	4	1.0 ± 0.1	3	1.3 ± 0.3	3	1.4 ± 0.2	3	1.0 ± 0.3	3	0.4 ± 0.1	**5**	16.4 ± 5.4^*^	**5**	58.1 ± 22.4^*^	3	1.9 ± 0.2
DHX9	4	1.1 ± 0.3	3	1.3 ± 0.1	3	1.8 ± 0.4	3	1.6 ± 0.2	3	0.8 ± 0.1	5	2.0 ± 0.3	5	1.0 ± 0.2	3	1.0 ± 0.1
**DHX36**	4	1.0 ± 0.1	3	1.4 ± 0.5	3	1.9 ± 0.3	3	1.3 ± 0.1	3	0.5 ± 0.2	5	1.0 ± 0.4	5	1.1 ± 0.3	3	0.7 ± 0.1
AIM	4	/	3	/	3	/	3	/	3	/	5	/	5	/	3	/
cGAS	4	1.0 ± 0.1	3	1.1 ± 0.0	3	2.4 ± 0.3	3	1.6 ± 0.1	3	1.0 ± 0.1	5	0.7 ± 0.2	5	1.2 ± 0.4	3	1.9 ± 0.2
**DAI/ZBP1**	4	1.0 ± 0.2	3	1.4 ± 0.4	3	1.4 ± 0.3	3	1.3 ± 0.1	3	0.5 ± 0.0	**5**	3.4 ± 0.1^*^	**5**	27.2 ± 1.8^*^	3	3.1 ± 0.3^*^
DDX41	4	1.0 ± 0.1	3	1.1 ± 0.2	3	1.3 ± 0.2	3	1.1 ± 0.1	3	0.7 ± 0.1	5	1.5 ± 0.3	5	1.4 ± 0.1	3	0.9 ± 0.1
LRRFIP1	4	1.1 ± 0.2	3	1.2 ± 0.3	3	1.8 ± 0.5	3	1.8 ± 0.1	3	0.8 ± 0.1	5	1.5 ± 0.1	**5**	2.7 ± 0.1^*^	3	1.0 ± 0.2
P202	4	1.0 ± 0.2	3	1.2 ± 0.2	3	1.5 ± 0.3	3	1.2 ± 0.2	3	0.9 ± 0.1	5	1.7 ± 0.5	**5**	3.7 ± 0.3^*^	3	0.9 ± 0.2
**P204**	3	1.0 ± 0.0	3	1.3 ± 0.2	3	1.0 ± 0.0	3	1.0 ± 0.1	3	1.1 ± 0.1	3	2.5 ± 0.7	**3**	2.6 ± 0.2^*^	3	1.3 ± 0.2
SOX2	3	1.0 ± 0.3	3	1.0 ± 0.3	2	1.0 ± 0.3	2	1.4 ± 0.1	2	0.4 ± 0.0	2	0.5 ± 0.1	2	0.5 ± 0.2	2	0.9 ± 0.2
MRE1	3	1.0 ± 0.2	3	0.9 ± 0.2	2	1.8 ± 0.6	2	0.9 ± 0.0	2	0.6 ± 0.0	2	1.1 ± 0.1	2	0.8 ± 0.1	2	1.0 ± 0.1
Ku70	3	1.0 ± 0.2	3	0.9 ± 0.1	2	1.9 ± 0.7	2	0.8 ± 0.1	2	0.8 ± 0.0	2	1.2 ± 0.1	2	0.7 ± 0.1	2	1.2 ± 0.0

Legend: cGAS, cyclic guanosine monophosphate-adenosine monophosphate synthase; DAI/ZBP1, DNA-dependent activator of interferon regulatory factor; DDX41, DEAD (Asp-Glu-Ala-Asp) box polypeptide 41; DDX60, DEAD (Asp-Glu-Ala-Asp) box polypeptide 60; DHX9, DEAH (Asp-Glu-Ala-His)box helicase 9; DHX36, DEAH (Asp-Glu-Ala-His) box helicase 36; LRRFIP1, leucine-rich repeat flightless-interacting protein 1; p204, interferon activated gene 204; p202, interferon activated gene 202; MRE11, meiotic recombination 11 homolog, double strand break repair nuclease; SOX2, SRY (sex determining region Y)-box 2; Ku70, Lupus Ku autoantigen protein p70^*^statistically significant difference compared to control group (*P* < 0.05); ND, not detected.

**Figure 3:**
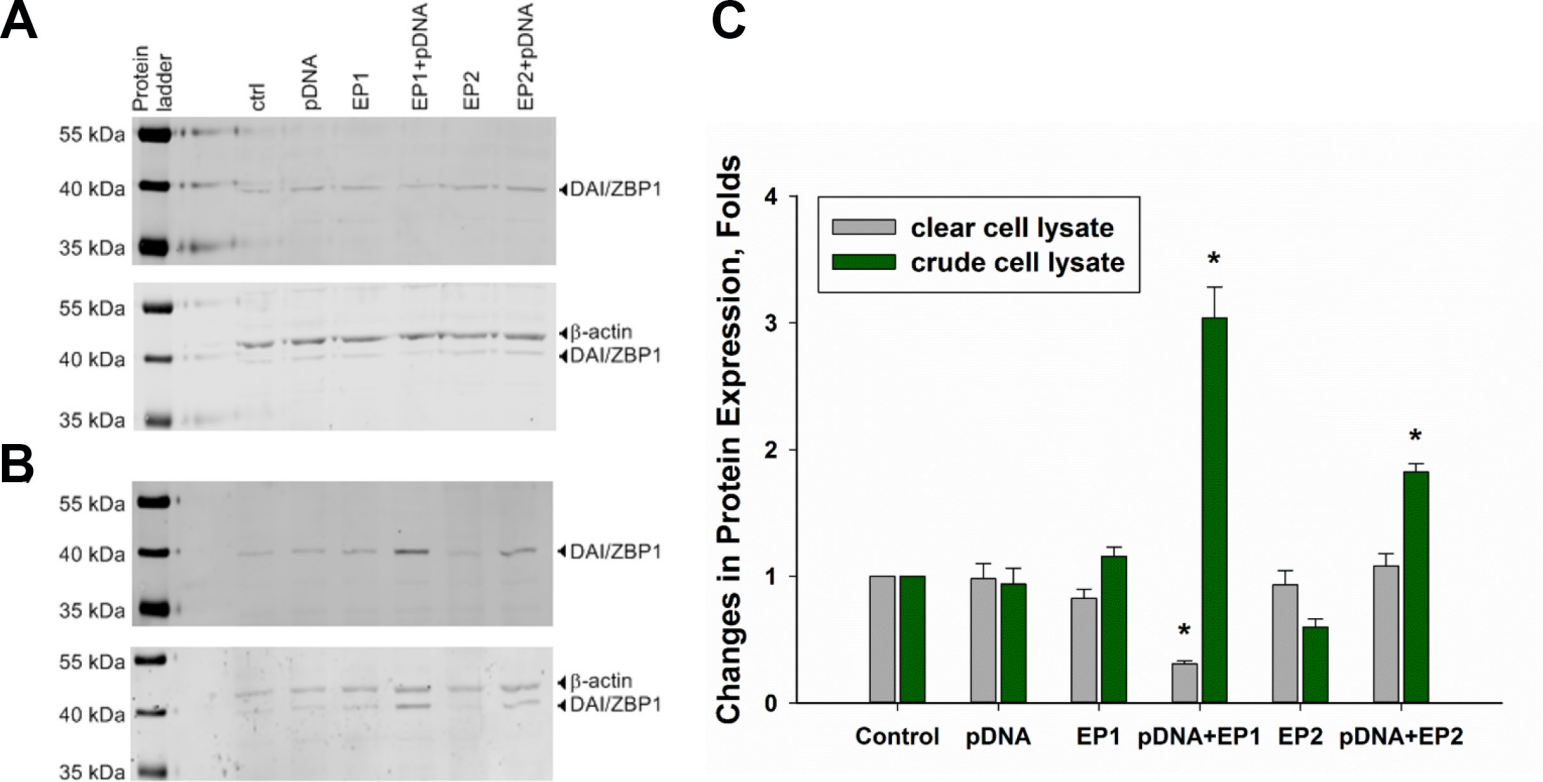
Changes in DAI/ZBP-1 expression in TS/A cells Panel (**A**) shows Western blot analysis of DAI/ZBP1 expression among different experimental groups when cell lysate was cleared by centrifugation, while panel (**B**) shows the analysis of crude cell lysate. Panel (**C**) represents the results of 3 independent experiments (mean ± standard error of the mean, *n* = 3; open bars represent clear lysate, filled ones represent crude lysate;^**^p < 0.01; ^*^p < 0.05).

Our previous study demonstrated that increased mRNA expression for particular PRRs can translate into increased protein levels in B16F10 melanoma cells *in vitro* [25]. Here, TS/A cells were exposed to electrical pulses using the EP1 or EP2 pulse protocols in the presence and in the absence of pDNA. When cleared cell lysates were analyzed by Western blotting, a significant decrease in DAI/ZBP-1 protein expression was observed in the EP1+pDNA experimental group (Figure 3A). This was quite unexpected, since mRNA levels for this protein were the most highly upregulated in this experimental group (Table 1).

Previous work from other groups showed that DAI/ZBP-1 resides in the cytosol with a diffuse, but partially granular-like pattern in HeLa and L929 cells [46, 47]. Overexpressed proteins may form inclusion bodies that could be eliminated from the cell lysate during centrifugation. So, the final step of centrifugation was excluded from the lysate preparation protocol and, when the crude lysate was analyzed by Western blotting, significant upregulation of DAI/ZBP1 protein was observed in EP1+pDNA and, to the lesser extent, in EP2+ pDNA experimental groups (Figure 3B, 3C).

### Increased expression of IFNβ and TNFα after electrotransfer

**Figure 4:**
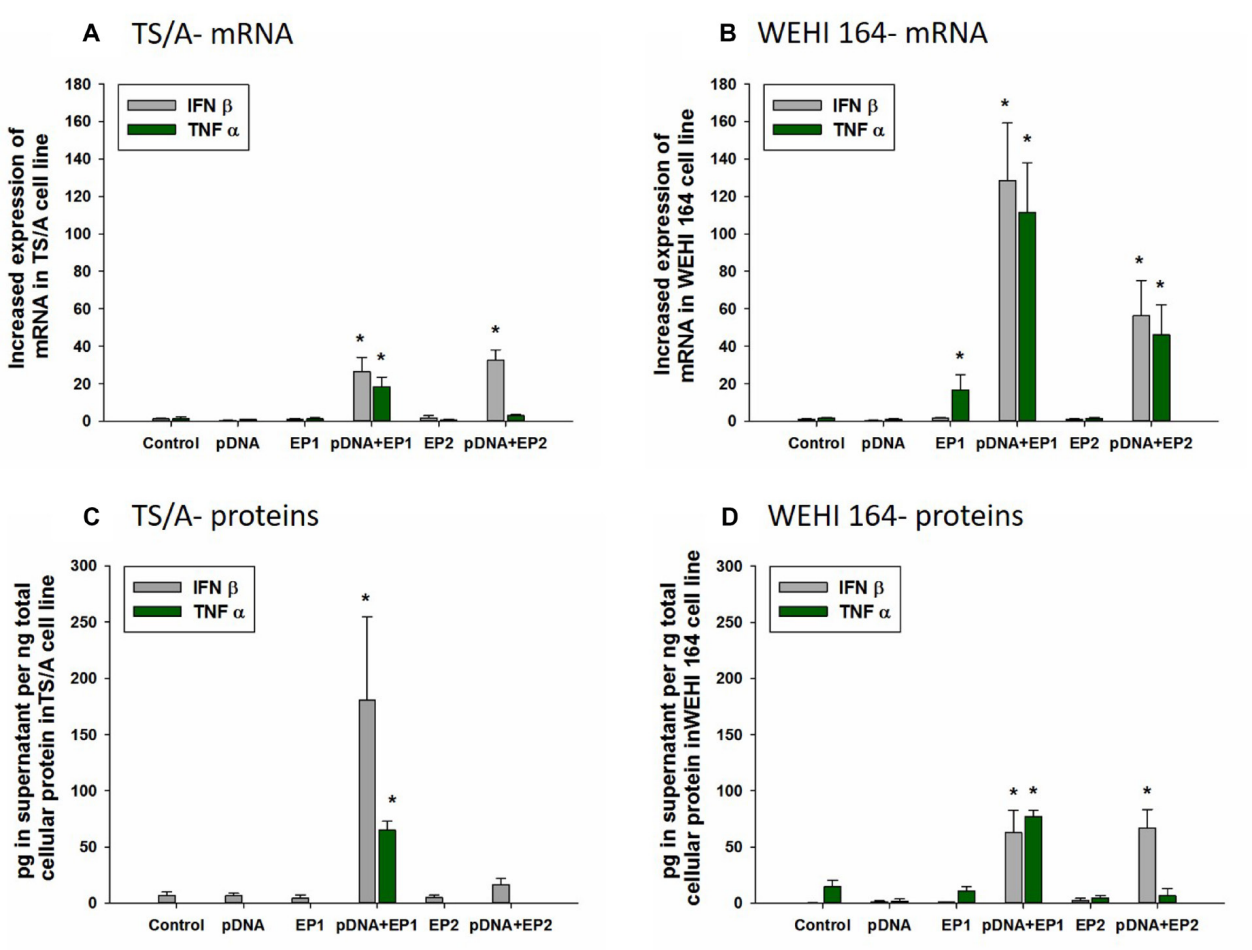
Effect of different electrotransfer pulse protocols on fold changes in mRNA and protein levels of INFβ and TNFα Levels of IFNβ and TNFα mRNA (**A**, **B**) were determined 4 hours after electrotransfer of vector pDNA using the pulse protocols described in methods and Figure 1. Intracellular levels of IFNβ and TNFα in the supernatant (**C**, **D**) were measured by ELISA 4 hours after electrotransfer.^*^statistically significant difference compared to electrotransfer protocol only (EP) groups (*p* < 0.05).

**Figure 5:**
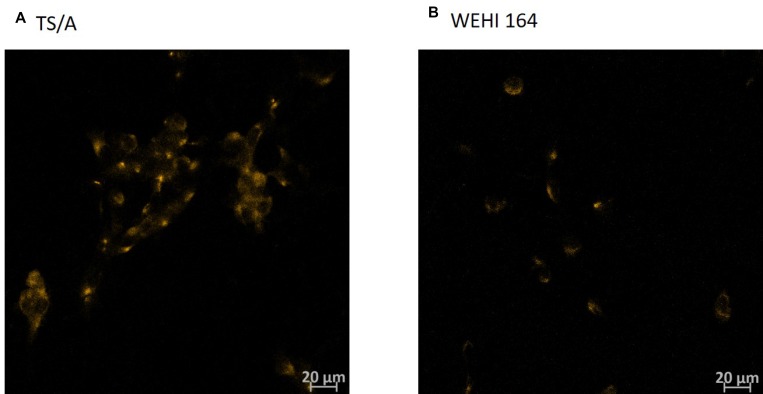
Immunohistochemistry of TNFR1 receptor in TS/A and WEHI 164 cell line Cells were fixed with 4% paraformaldehyde (**A**, **B**). Cells where receptor for TNFα (TNFR1) was present were stained in orange due to the Cy3-conjugated Donkey Anti-Rabbit IgG.

In each group receiving electrotransfer of pDNA, a marker for DNA sensor activation, IFNβ, was upregulated on the mRNA level in both cell types. (Figure 4A, 4B) In TS/A cells, this upregulation of approximately 30-fold was comparable between the two pulse protocols (Figure 5A). However, this increase was reflected in increased protein levels only after pDNA delivery using EP1 (Figure 4C). IFNβ upregulation was more marked in WEHI 164 cells (Figure 4B). In these cells, the increase in IFNβ protein levels, approximately 150-fold, was similar between the two pulse protocols (Figure 4D).

The mRNA of the inflammatory marker TNFα was upregulated and increased production of protein detected after electrotransfer of pDNA. Specifically, in TS/A cells, the increase in mRNA and protein levels were observed only after electrotransfer of pDNA using EP1 pulse protocol (Figure 4A, 4B). In WEHI 164 cells, the mRNA of TNFα was upregulated after electrotransfer of pDNA with each pulse protocols (Figure 4B); however, production of TNFα protein was increased only after pDNA electrotransfer using EP1 pulse protocol (Figure 4D).

### Detection of mRNA IFNβ receptor (IFNAR1) and detection of TNFα receptor (TNFR1) by Immunohistochemistry in TS/A and WEHI 164 cell lines

Type I interferon receptors are ubiquitously expressed in cells [48], which was confirmed with RT-PCR on the mRNA level in both cell lines (data not shown). Immunocytochemical staining of cells also confirmed that both cell lines express TNFα receptor (Figure 5B).

## DISCUSSION

The results of our study demonstrate that, in general, different gene electrotransfer pulse protocols that have different transfection efficiencies and produce different levels of immediate cell death can have similar effect on the expression of cytosolic DNA sensors in tumor cell lines. Although higher transfection efficiency was obtained in WEHI 164 cells, similar sensors (DAI/ZBP1, DDX60 and p204) were upregulated in two cell lines TS/A and WEHI 164 as in our previous study on B16F10 melanoma cells [49]. The EP1 protocol produced higher levels of IFNβ and TNFα in both cell lines than the EP2 protocol; these levels were higher in WEHI 164 than in TS/A cells. Finally, cell death can be induced by the electrotransfer of pDNA, with apoptosis prevalent in WEHI 164 after delivery with the EP2 protocol and necrosis prevalent after delivery with the EP1 protocol, while in TS/A cells both types of cell death occurred to the same level.

We initially determined transfection efficiency. Delivery with the EP2 pulse protocol led to a high percentage of transfected cells of both cell lines, while pDNA delivery with the EP1 pulse protocol significantly increased only the median fluorescence intensity in WEHI 164 cells. This indicated that higher number of plasmid copies were introduced into the cells. Both types of pulse protocols were previously used in many studies *in vitro* and *in vivo* [44, 50–52]. *In vivo,* in B16F10 mouse melanomas and P22 rat carcinomas, both pulse protocols yielded similar GFP expression, while in other tumor models (T24 human bladder carcinoma, SaF mouse sarcoma), the EP1 pulse protocol resulted in higher expression [53]. Many parameters pertinent to the tumor type, to the tumor microenvironment, to the pDNA and to the electrical parameters influence the transfection efficiency [17, 54–57]. Previous studies using a combination of short, high voltage and long, low voltage pulses showed a similar or even greater transfection efficiency *in vivo* in tissues compared to electrotransfer with EP1 and EP2 [16, 55, 58, 59]. *In vitro*, the transfection efficiency using EP3 pulse protocol in Chinese hamster ovary cells was similar to that obtained with high voltage alone [66]. In contrast, our results showed that this combined pulse protocol was not effective for *in vitro* transfection of WEHI 164 fibrosarcoma or TS/A carcinoma cells, although modifications of this pulse protocol may be more successful. The EP3 pulse protocol used in our study had the lowest effect on transfection efficiency (<5%), DNA sensor mRNA upregulation (<2-fold) and cell survival (>80%) of the pulse types tested. This pulse protocol clearly delivers DNA more effectively to cells in an *in vivo* environment [16, 58, 60, 61]. In *in vivo* environments, high voltage pulses are crucial for efficient permeabilization of the membrane which enables transfection, while low voltage pulses provide electrophoretic force to pull negatively charged DNA molecules toward the cell membranes [58, 60]. This was demonstrated for skin and muscle, while for tumors the separation of electrophoretic and permeabilization components did not result in improved transfection [16, 55]. The same seems true for the selected cell lines, since the combination pulse regimen triggered only minimal DNA entry into the cell.

The higher transfection efficiency after delivery with the EP2 protocol indicated more efficient DNA uptake by an endocytosis-like mechanism [21, 22]. In TS/A cells, this higher transfection efficiency did not correlate with the upregulation of PRR mRNAs; both pulse protocols upregulated DDX60 and DAI/ZBP1, while the EP1 protocol additionally upregulated DHX36, and the EP2 protocol additionally upregulated p204. On the other hand, a correlation between higher transfection efficiency of EP2 protocol and larger number of upregulated cytosolic DNA sensors was obtained in WEHI 164 cells. Delivery with pulse protocol EP3, combining one high voltage pulse with four low voltage pulses, only modestly transfected cells, had no effect on cell death and only minimally upregulated p204 in TS/A cells and DAI/ZBP1 in WEHI 164 cells. The possible explanation for these observations could be different entry mechanisms for pDNA [28]. During electrotransfer, DNA enters the cell via an endocytosis-like mechanism [21, 22] but must escape the endosomes through the cytosol to the nucleus to be expressed. Early endosomal escape from the endosome to the nucleus can also occur [26]. Alternatively, DNA may be delivered directly to the cytosol through electropores formed in the plasma membrane [27, 28]. Thus, due to the high transfection efficiency, higher cell survival, and pronounced upregulation of DNA sensors, the entry of pDNA with EP2 protocol is most probably endocytosis, while DNA entry following EP1 is more likely to be through electropores, although other groups demonstrated that endocytosis occurs after the use of EP1 pulses too [21, 22].

The pulse protocols used in our study were cytotoxic to both cell lines, even in the absence of pDNA. The most cytotoxic was EP1 protocol, followed by EP2 protocol, while EP3 only minimally reduced cell survival of TS/A cells. Nanosecond pulses can directly induce apoptosis through activation of mitochondrial signal pathways [62]. Alternatively, the destruction of the cell membrane by subnanosecond electric pulses can lead to necrosis [63]. Electric pulses with appropriate parameters can kill cancer cells directly through the induction of apoptosis without chemical drugs [64]. In both cell lines, application of EP1 pulses were highly cytotoxic even in the absence of pDNA. This electrical cell damage is probably due to the too high electric fields that cause membrane damage resulting in electrolyte imbalance, influx of water, osmotic swelling of the cells and consequently cell death by necrosis [63, 65, 66]. Molecular dynamics simulations of electroporation in several lipid systems demonstrated that direct interaction between electric field and phospholipids exist [67]. Furthermore, in CHO cells, a direct interaction between the movement of membrane phosphatidylserine and electric field was demonstrated [68]. Thus, we can presume that the induction of necrosis following EP1 protocol is a physical process. Interestingly, CHO cells retained high viability using this pulse protocol, emphasizing the variation between cell lines [69].

Enhanced toxicity of the electric pulses in presence of the pDNA was reported in previous studies [70, 71]. In support of these observations, the cell death mechanism in response to the combination of pulses and DNA was both pulse protocol and pDNA dosage dependent (Figure 2), which is in agreement with our previous study [72], although there are differences in cell survival between the cell lines. Apoptosis was the major mechanism of cell death using the EP2 pulse protocol, while necrosis predominated when using the EP1 pulse protocol in WEHI 164 cells. In TS/A cells, both apoptosis and necrosis contributed equally to cell death. The cell morphology, which was assessed six hours after the therapy, demonstrated mainly necrotic cells death, while the data obtained by flow cytometry 20 h after therapy, demonstrated that both types of cell death occurred. This difference is due to the fact that apoptosis is a longer process than necrosis.

Cell death can also occur due to the loss of ATP after EP [73]. A significant reduction in intracellular ATP immediately after EP was demonstrated. This reduction was higher after the application of the EP1 pulse protocol than after the EP2 protocol. This reduction might indicate that more membrane damage was produced using long, low voltage pulses (EP1 pulse protocol) than short, high voltage pulses (EP2) (Figure 2). However, although the ATP loss was significant, it did not correlate with the reduction of cell survival, thus the obtained cell death could not be a direct consequence of the ATP loss that occurred within 30 minutes of electrotransfer.

The presence of foreign DNA in the cell can be detected by DNA sensors located in the cytosol or on the endosomal membrane. The activation of cytosolic DNA sensors takes place when these sensors detect and bind DNA in the cytosol [29–33]. Upon activation, these sensors induce the production of cytokines and potentially cell death. The upregulation of both endosomal and cytosolic DNA sensors was explored in our study. The results demonstrate that effective electrotransfer protocols have similar effects on the expression of DNA sensors, induce a cytokine response and produce cell death *in vitro*. Similar cytosolic DNA sensors were upregulated in TS/A murine mammary adenocarcinoma and WEHI 164 fibrosarcoma cells after effective electrotransfer protocols EP1 and EP2, confirming our previous study on B16F10 melanoma [72]. These results imply that many tumor cell types respond similarly to pDNA electrotransfer.

Although, in general, the basal levels of mRNA of the assayed PRRs were higher in TS/A cells, the upregulation was more pronounced in WEHI 164 cells (data not shown). The upregulation of DAI/ZBP1, DDX60, and p204 was detected previously in B16.F10 melanoma cells, and this was confirmed in TS/A mammary carcinoma cells. Basal levels of p204 mRNA were higher than those of DAI/ZBP1 and DDX60; however, the upregulation was more dramatic for DAI/ZBP1 and DDX60. This is similar to the results obtained by Zhu in Leydig cells, indicating that p204 may be the first sensor to respond to presence of DNA and that foreign DNA in the cytosol pronouncedly increased the expression of additional DNA sensors [74]. Interestingly, additional mRNAs were minimally yet significantly upregulated in WEHI 164 fibrosarcoma cells. Specifically, after delivery with the EP2 pulse protocol, LRRFIP1 and p202 were upregulated. Similar to DDX60 and DAI/ZBP1, these proteins are also known to activate IFNβ [75, 76]. Indeed, IFNβ mRNA and protein, markers for DNA sensor activation, were upregulated after pDNA electrotransfer. Following EP1 delivery, protein levels were similar between the cell types, while only WEHI 164 cells responded to delivery with EP2. Nearly every cell type expresses IFN receptor 1, IFNAR1 [48, 77], and as expected, both cell types express the mRNA for IFNRA1 (data not shown). This potentiates the possible autocrine-paracrine effect, producing cell death.

Furthermore, upregulation of TNFα mRNA was observed in TS/A cells after DNA electrotransfer using EP1 pulse protocol or in WEHI 164 cells after any DNA electrotransfer or at low levels after exposure of cells to electric pulses alone. In WEHI 164 cells, mRNA levels did not consistently correlate to increased protein. TNFα protein was detected in either cell type only after delivery with EP1. While mRNA levels can predict protein levels, a lack of correlation is common [78].

Upregulation of DAI/ZBP1 mRNA in TS/A cells translated into upregulation on the protein level. The expression of DAI/ZBP1 was significantly increased after pDNA delivery with the EP1 protocol, and, to the lesser extent, the EP2 protocol when crude lysate containing all the cellular components was analyzed. Unexpectedly, the results obtained from cleared lysate containing only soluble proteins demonstrated a significant decrease of DAI/ZBP1 content after pDNA delivery with EP1 pulse protocol. These results lead to the suggestion that after pDNA delivery with the EP1 pulse protocol, which produces the lowest cell survival rate among the pulse protocols we applied, DAI/ZBP1 protein becomes so abundant that it forms insoluble inclusion bodies as overexpressed proteins do in many cases under unfavorable conditions. This suggestion leaves the open question of why the amount of DAI/ZBP1 is significantly decreased in clear extract after pDNA delivery with the EP1 pulse protocol. One possible explanation could be that after inclusion body formation, the expression of this protein slows, while protein molecules still present in the cytoplasm become less abundant due to common protein degradation processes. Additional study is required to investigate this possibility.

**Figure 6:**
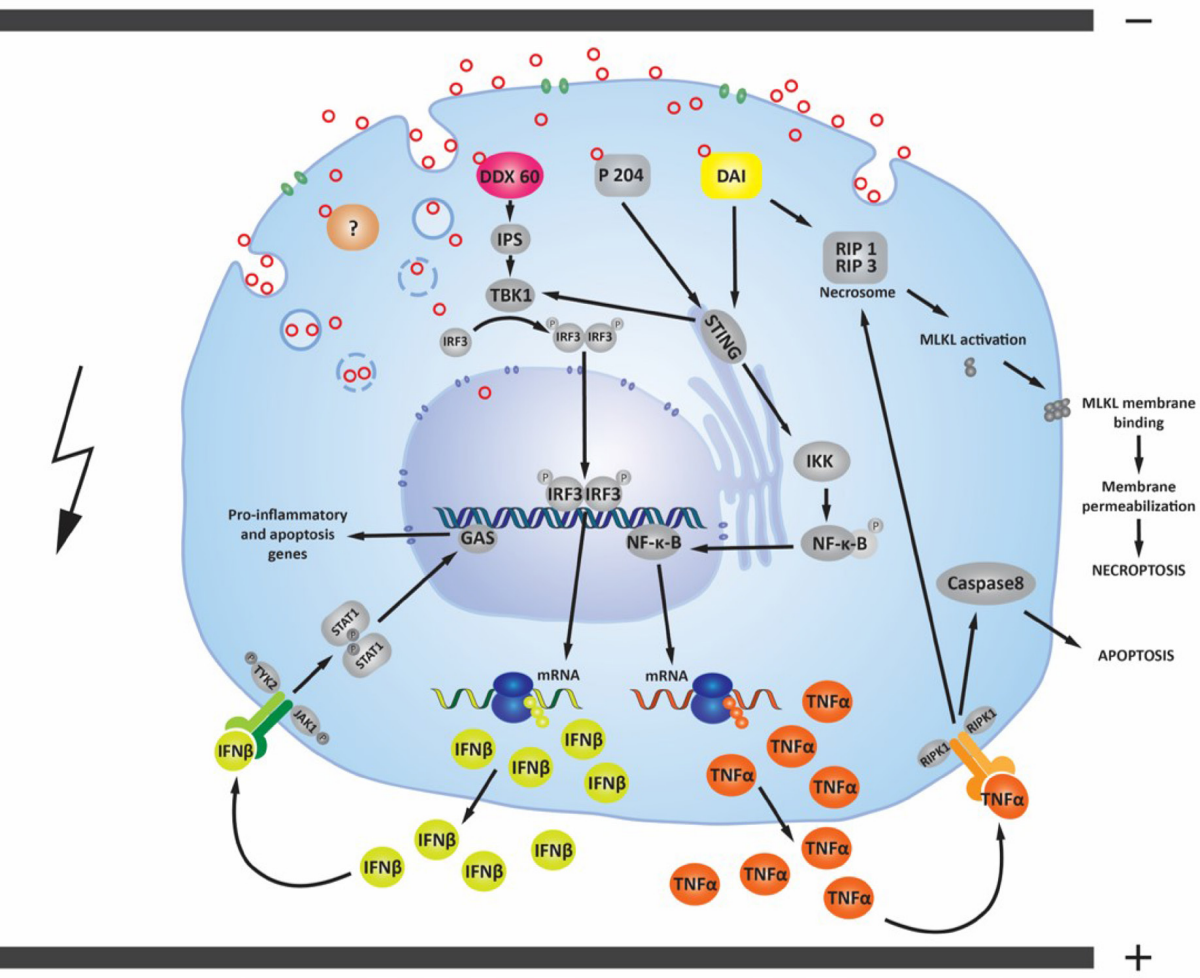
Possible signaling pathways activated by pDNA electrotransfer of tumor cells After electrotransfer, pDNA (red circles) primarily enters the cell through endocytosis (blue circles). Plasmid DNA must undergo endosomal escape to enter the cytosol. Endosomal escape can occur early in the endocytosis process or later, before pDNA enters the nucleus [21, 26]. pDNA may possibly enter the cytosol directly via electropores formed in cell membrane [28]. The presence of pDNA inside the cytosol of different tumor cell lines is associated with the upregulation of cytosolic DNA sensors DDX60, DAI/ZBP1 and the p204 [44, 73, 74]. If these cytosolic DNA sensors are activated by binding pDNA, the transcription factor interferon regulator factor 3 (IRF3) is transported from the cytosol to the nucleus to induce transcription of IFNβ and other genes [29, 47, 82, 98]. The upregulated and secreted IFNβ protein may bind to its cell-surface receptor(s) in an autocrine or paracrine fashion, leading to transcription of pro-inflammatory and apoptotic genes. The DNA sensors p204 and DAI/ZBP1 can activate a signaling pathway which leads to the nuclear translocation of the transcription factor NF-ĸB and consequently to transcription of pro-inflammatory cytokines, such as TNFα, which is secreted from the cell [29, 47, 80, 81, 99]. TNFα may bind to cell-surface receptors, which leads to the activation of two different death mechanisms (apoptosis or necroptosis) [90]. Another signaling pathway of the cytosolic DNA sensor DAI/ZBP1 leads directly to necroptosis through MLKL activation [100]. Cytosolic DNA sensors and their signaling pathways described here are present and upregulated in mouse TS/A adenocarcinoma cells, mouse WEHI 164 fibrosarcoma cells, and mouse B16F10 melanoma cells [25]. Other DNA sensors, whether upregulated, not upregulated, or as yet undiscovered (labeled with “?”) may also influence these signaling pathways.

Upregulation of p204, DAI/ZBP1 and DDX60 activate signaling pathways that overlap and result in cytokine production that can lead to both apoptosis and necrosis [29, 79–83] (Figure 6). The cell death after electrotransfer of pDNA using the EP1 pulse protocol could be a consequence of the production of IFNβ and TNFα. TNFα has shown an antitumor effect on tumor cells or tumors in combination with other treatments [84–88]. Cell lines that possess receptors for INFβ and/or TNFα and receptor binding can activate necroptosis or apoptosis [89, 90]. Necroptosis is a programmed form of necrosis that requires the proteins RIPK3, MLKL, DAI/ZBP1 and it is induced by death receptors, interferons, toll-like receptors, intracellular RNA and DNA sensors [91, 92]. Otherwise, the features of necroptosis such as disruption of cell membrane and loss of organelles are the same as necrosis. Therefore, these death processes cannot be morphologically distinguished. To confirm that TS/A and WEHI 164 cells might respond in an autocrine or paracrine action to the production of IFNβ and TNFα proteins, the presence of IFN receptor (IFNAR1) and TNFα receptor (TNFR1) were confirmed in both cell lines. In TS/A cells, cell death due to the combination of apoptosis and necrosis was observed, in WEHI 164 cells, a higher level of necrosis was observed after DNA delivery with both pulse protocols. Binding of proteins IFNβ and TNFα to their receptors could contribute to different cell death mechanisms after DNA electrotransfer.

## MATERIALS AND METHODS

### Cell lines

TS/A murine mammary adenocarcinoma cells [93] were cultured in advanced minimum essential medium (AMEM, Gibco, Thermo Fisher Scientific, Waltham, MA, USA) supplemented with 5% fetal bovine serum (FBS, Gibco) in 5% CO_2_ humidified atmosphere at 37° C. The cell line was tested for authentication in 2017 at IDDEx Bioresearch laboratory. WEHI 164 murine fibrosarcoma cells (ATCC CRL-1751, American Type Culture Collection, Manassas, VA, USA) were cultured in Roswell Park Memorial Institute Medium (RPMI-1640, Gibco) supplemented with 5% fetal bovine serum (FBS, Gibco) in 5% CO_2_ humidified atmosphere at 37° C.

### Plasmids

The vector plasmid gWiz Blank was commercially prepared (Aldevron, Fargo, ND, USA) at concentration of 2 mg/ml in physiological saline. Additionally, concentrations of 1 mg/ml by further dilution and 3.5 mg/ml by concentration (Concentrator plus, Eppendorf, Hamburg, Germany) were prepared.

Plasmid EGFP-N1 (pEGFP-N1, BD Biosciences, San Jose, CA, USA), encoding green fluorescent protein, was used for transfection efficiency experiments. It was isolated after amplification in a competent *Escherichia coli* (TOP10; Thermo Fisher Scientific) using Maxi-Endo Free Plasmid Kits (Qiagen, Hilden, Germany) according to the manufacturer’s instructions. The concentration of isolated plasmid was measured with Epoch Microplate Spectrophotometer (BioTek Instruments, Winooski, VT, USA).

### DNA Electrotransfer pulse protocols

Cells were prepared and processed as previously described [94]. Fifty µL containing 20 µg pDNA per 1 × 10^6^ cells was pipetted between two electrodes with 2 mm gap and three different electric pulse protocols were applied: EP1 (8 pulses, 600 V/cm, 5 ms duration at frequency 1 Hz), EP2 (6 pulses, 1300 V/cm, 100 µs duration at frequency 4 Hz) and EP3 (1 pulse 600 V/cm, 100 µs duration + 4 pulses 80 V/cm, 100 ms duration, frequency 1Hz). The cells were then incubated in AMEM or RPMI in 6-cm Petri dishes (Corning Incorporated, Corning, NY, USA) for determination of transfection efficiency, Adenosine triphosphate (ATP) leakage and cell death mechanism and in ultralow attachment 6- well or 24-well plates (Corning Incorporated) for cell survival assays, determination of morphological changes and expression of DNA sensors and selected cytokine mRNA and protein.

### Transfection efficiency

Two days after electrotransfer with 10 µg pEGFP-N1 per 1 × 10^6^ cells, the cells were imaged by fluorescence microscopy (Olympus IX-70, Hamburg, Germany), then trypsinized and resuspended in 400 µl of PBS for flow cytometry analysis (FACSCanto II flow cytometer, BD Biosciences). A 488-nm laser (air-cooled, 20 mW solid state) and 530/30-nm band-pass filter were used for the excitation and detection of green fluorescent protein fluorescence, respectively. A total of 20,000 events were measured. The percentage of transfected cells represented transfection efficiency, while the fluorescence intensity was determined as an indirect measure of the amount of pDNA that was introduced into the cells [95].

### Cell survival assay

Cell survival was determined 72 hours after electrotransfer [96] of 10 µg, 20 µg, and 35 µg gWiz Blank per 1 × 10^6^ cells. After electrotransfer, 1 × 10^3^ cells were incubated in 0.1 ml AMEM in 96-well plates (Corning Incorporated) at 37° C in a 5% CO_2_ humidified incubator. The viability of the cells after pulse delivery was determined using Presto Blue (Thermo Fisher Scientific) per manufacturer’s instructions and normalized to the viability of control cells that were not exposed to electric field.

### ATP determination assay

Thirty minutes after electrotransfer of gWiz Blank, 1 × 10^6^ cells were incubated in 1 ml of appropriate media for 1h at 37° C at 5% CO_2_. The cells were centrifuged (Heraeus Fresco 21 centrifuge, Thermo Fisher Scientific) for 10 minutes at 12000 g. The supernatant was removed, and the cell pellets were resuspended in 1 ml of boiling distilled water, vortexed, immediately placed on ice, and centrifuged for 5 minutes at 4° C and 21000 g. The samples were transferred to the white 96-well plates and ATP content was determined (ATP determination kit, Molecular Probes, Thermo Fisher Scientific) according to manufacturer’s instructions.

### Expression of PRRs and selected cytokines

Four hours after gWiz Blank electrotransfer with pulse protocols EP1, EP2 and EP3, total RNA was isolated from 1 × 10^6^ cells, cDNA was synthesized and diluted 1:10. Relative mRNA levels were determined with quantitative RT-PCR using IDT Oligonucleotides (IDT, Coralville, IA, USA) (Supplementary Table 1) and Syber Select Maste Mix (Thermofisher scientific).

### ELISA

Four hours after electrotransfer with gWiz Blank, cell cultures were washed with PBS, lysed (Mammalian Protein Extraction Buffer, GE Healthcare, Piscataway, NJ, USA), and total protein quantified by BCA assays (Pierce Biotechnology, Rockford, IL, USA). IFNβ was measured by ELISA (PBL Assay Science, Piscataway, NJ, USA) in normalized cell culture lysates. Tumor necrosis factor α (TNFα) was measured by ELISA (Ray Biotech, Norcross, GA, USA) in cell culture supernatants and then normalized to the total protein levels in the lysates to account for differences in cell number and viability.

### Western blot analysis

TS/A cells were electrotransfected with gWiz Blank plasmid as described above using EP1 and EP2 pulse protocols. The cells were then seeded into low-attachment 6-well plates (Corning Incorporated, Corning, NY, USA) and incubated for 9 hours. After incubation, the cells were lysed using RIPA buffer in accordance with the manufacturer’s protocol (Santa Cruz Biotechnology Inc., Dallas, TX, USA). For the preparation of crude lysate, the final centrifugation step was excluded. Total protein content was determined using a Pierce BCA Protein Assay Kit (Fisher Scientific) then adjusted with RIPA buffer. Twenty-five µg or 40 µg of total protein (for cleared and crude lysate, respectively) per well was separated in a 10% polyacrylamide gel, transferred onto nitrocellulose membrane (Bio-Rad, Hercules, CA), blocked for 1 hour in 5% milk in TBS Tween-20 buffer (Fisher Scientific) and incubated overnight at 4° C with primary antibodies: rabbit anti-DAI/ZBP1 or rabbit anti-β-actin as a loading control (Fisher Scientific). After washing with TBS Tween-20 buffer, the membrane was probed with Alexa Fluor 680 goat anti-rabbit secondary antibody (Fisher Scientific) for 45 min at room temperature, washed and protein bands were visualized with Odyssey Infrared Imaging System (LI-COR, Inc., Lincoln, NE, USA). The band intensity was quantified using Image J software [97].

### Morphological changes of tumor cells after DNA electrotransfer

Six hours after electrotransfer of gWiz Blank, 1 × 10^3^ cells in 80 μl of PBS were transferred to a slide chamber and centrifuge at 123 g for 4 minutes (Cytospin 2, Thermo Shandon, Runcorn, UK). The slides were air dried then stained with Giemsa’s Azure methylene blue solution (Merck, Darmstadt, Germany) according to the manufacturer’s protocol. Images of cell morphological changes were captured with a DP72 CCD camera using Olympus BX-51 microscope (Olympus, Tokio, Japan). Necrotic cells were characterized as eosinophilic cells or as cell ghosts without the presence of nucleus, while apoptotic cells characteristics included cytoplasmic shrinkage, nuclear condensation, nuclear fragmentation and the formation of apoptotic bodies.

### Determination of cell death mechanisms

Based on our previous results [49], cell death mechanism was determined twenty hours after electrotransfer of gWiz Blank by FITC Annexin V Apoptosis Detection Kit with 7-AAD (7-Aminoactinomycin D) (BioLegend, San Diego, CA, USA) according to manufacturer’s instructions for flow cytometric analysis (FACSCanto II, BD Biosciences). A total of 20,000 events were measured. Apoptosis was evaluated by phosphatidylserine detection in the outer plasma membrane leaflet using Annexin V. Necrotic cells were detected with 7-AAD, which has a high DNA-binding constant and can pass into the nucleus and bind to DNA in necrotic cells.

### Immunohistochemistry of TNFR1 receptor

1 × 10^5^ cells were plated in each well of µ-Chamber 12 well glass slides (Ibidi, Munich, Germany). After 24 hours the cells were fixed with 4% paraformaldehyde. Then, the cells were stained immunohistochemically using primary Anti-TNF Receptor I antibody (Abcam, Cambridge, MA, USA) and Cy3-conjugated AffiniPure Donkey Anti-Rabbit IgG as a secondary antibody (Jackson Immuno Research, PA, USA).

### Statistical analysis

For most experiments, statistical analysis was performed by SigmaPlot 12.0 (Systac Software Inc., San Jose, CA, USA). Statistical evaluation was made by one-way analysis of variance (one-way ANOVA). Western blot results were analyzed by one-way ANOVA with Holm-Sidak test. A *p*-value of less than 0.05 was considered statistically significant. ELISA results were analyzed by two-way ANOVA with Tukey-Kramer multiple comparisons test (GraphPad Software, La Jolla, CA, USA). A *p*-value less than 0.05 was considered significant.

## CONCLUSIONS

We have shown presence of cytosolic DNA sensors in different tumor cell lines and the upregulation of several sensors after pDNA electrotransfer. This upregulation correlates with the expression of IFNβ and TNFα genes and proteins. The expression of cytosolic DNA sensors is pulse protocol dependent, as is the mechanism of cell death. These effects may be due to both electrical cell damage, which occurs immediately after application of electric pulses, and to the induction of apoptosis or necrosis through activation of cytosolic DNA sensors.​

## SUPPLEMENTARY MATERIALS FIGURES AND TABLES





## Abbreviations

AIM2: Absent in melanoma 2
ATP: Adenosine triphosphate
cGAS: Cyclic guanosine monophosphate-adenosine monophosphate synthase
DAI/ZBP1: DNA-depended activator of interferon regulatory factor/Z-DNA binding protein 1
DDX41: DEAD (Asp-Glu-Ala-Asp) box polypeptide 41
DDX60: DEAD (Asp-Glu-Ala-Asp) box polypeptide 60
DHX36: DEAH (Asp-Glu-Ala-His) box helicase 36
DHX9: DEAH (Asp-Glu-Ala-His) box helicase 9
ECT: Electrochemotherapy
ELISA: Enzyme-linked immunosorbent assay
EP: Electroporation
EP1: Electroporation pulse protocol one (8 pulses, 600 V/cm, 5 ms duration at frequency 1 Hz)
EP2: Electroporation pulse protocol two (6 pulses, 1300 V/cm, 100 µs duration at frequency 4 Hz)
EP3: Electroporation pulse protocol three (1 pulse 600 V/cm, 100 µs duration + 4 pulses 80 V/cm, 100 ms duration, frequency 1Hz)
IFNAR1: Interferon alpha/beta receptor 1
IFNβ: Interferon β
Ku70: Lupus Ku autoantigen protein p70
LRRFIP1: Leucine-rich repeat flightless-interacting protein 1
MRE11: Meiotic recombination 11 homolog, double strand break repair nuclease
ND: Not detected
p202: Interferon activated gene 202
p204: Interferon-inducible protein 204
pDNA: Plasmid DNA
pEGFP-N1: Plasmid for enhanced green fluorescent protein
PRR: Pattern recognition receptors
RIG I: Retinoic acid inducible gene I
RT-PCR: Real time reverse transcription polymerase chain reaction
SOX2: SRY (sex determining region Y)-box 2
TLR9: Toll-like receptor 9
TNFR1: Tumor necrosis factor receptor 1
TNFα: Tumor necrosis factor α
