# Supplementary material for: Tumor cell death after electrotransfer of plasmid DNA is associated with cytosolic DNA sensor upregulation
Source: Oncotarget. 2018 Apr 10;9(27):18665–81. doi: 10.18632/oncotarget.24816 (PMC5922346; doi:10.18632/oncotarget.24816)
Supplement: Supplementary file 1 [file oncotarget-09-18665-s001.pdf]

Tumor cell death after electrotransfer of plasmid DNA is associated with cytosolic DNA sensor upregulation

SUPPLEMENTARY MATERIALS

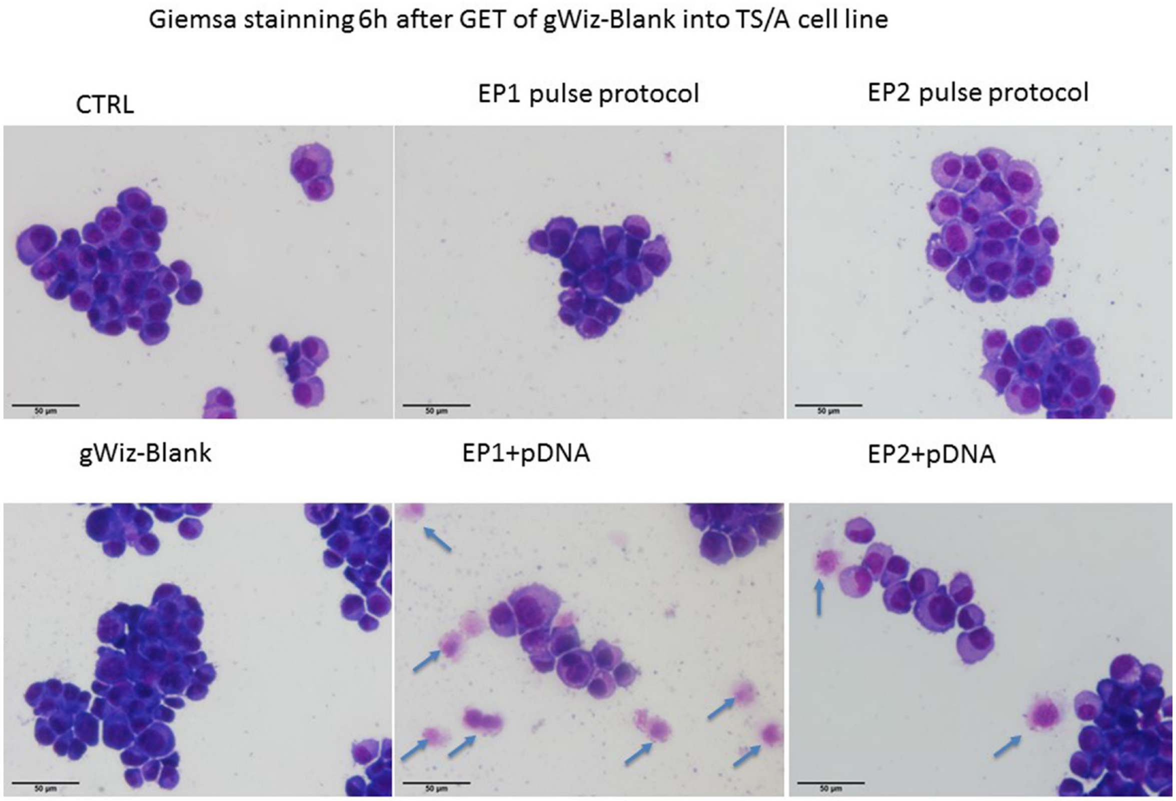

Supplementary Figure 1: Morphology of TS/A cell line 6 hours after pDNA electrotransfer. Blue arrows indicate necrotic cells displaying cytoplasmic fragmentation and only an outline of the nucleus.

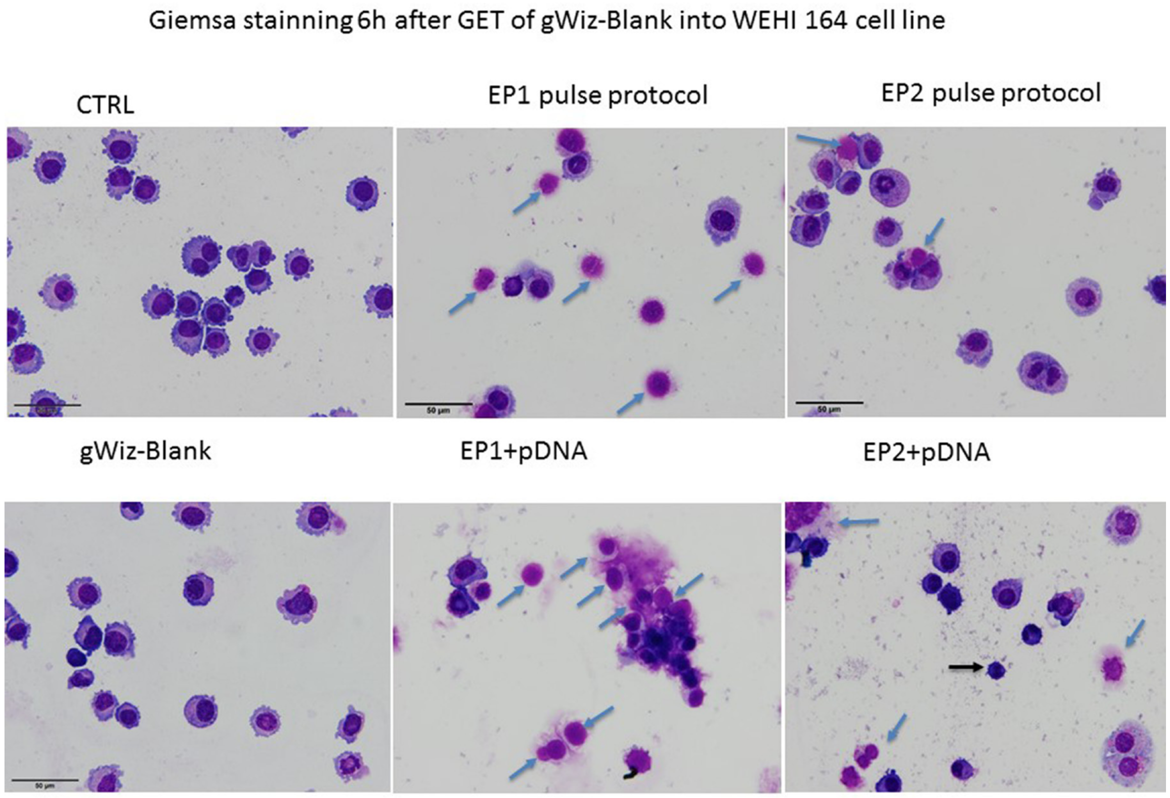

Supplementary Figure 2: Morphology of WEHI 164 cell line 6 hours after pDNA electrotransfer. Blue arrows indicate necrotic cells displaying cytoplasmic fragmentation and only an outline of the nucleus. Black arrows indicate apoptotic cells with vacuolization of the cytoplasm and formation of apoptotic bodies.

Supplementary Table 1: RT-PCR Information. See Supplementary_Table_1

Supplementary Table 2: Raw data for ATP leakage assay for TS/A and WEHI 164 cells

	**control**		**gWiz Blank**		**EP1**		**EP2**		**pDNA+EP1**		**pDNA+EP2**	
	**a.u. (mean ± SE)**	***n***	**a.u. (mean ± SE)**	***n***	**a.u. (mean ± SE)**	***n***	**a.u. (mean ± SE)**	***n***	**a.u. (mean ± SE)**	***n***	**a.u. (mean ± SE)**	***n***
TS/A	4833.3 ± 98.0	6	3325.9 ± 420.4	6	679.4 ± 23.6	9	1178.1 ± 304.5	6	154.4 ± 28.7	9	976.0 ± 90.4	6
WEHI 164	1404.3 ± 497.4	5	1276.2 ± 413.6	5	310.0 ± 61.3	5	713.2 ± 221.6	5	106.0 ± 20.7	4	456.0 ± 128.7	7

a.u. - arbitrary units; SE – standard error of the mean.

REFERENCES

1. Livak KJ, Schmittgen TD. Analysis of relative gene expression data using real-time quantitative PCR and the 2(-Delta Delta C(T)) Method. Methods. 2001; 25:402–8.
