# Supplementary material for: Tumor cell death after electrotransfer of plasmid DNA is associated with cytosolic DNA sensor upregulation
Source: Oncotarget. 2018 Apr 10;9(27):18665–81. doi: 10.18632/oncotarget.24816 (PMC5922346; doi:10.18632/oncotarget.24816)
Supplement: Supplementary file 2 [file oncotarget-09-18665-s002.docx]

**supplementary Table 1:**

RT-PCR Information.

| Experimental design | | | | | | | |  | |  | | |  | |  | |  |
| --- | --- | --- | --- | --- | --- | --- | --- | --- | --- | --- | --- | --- | --- | --- | --- | --- | --- |
| Definition of experimental and control groups | | | | | | | | Control group | | Cells incubated in selected media | | | | | | |  |
|  |  |  |  |  |  |  |  | pDNA | | Cells and plasmid DNA incubated in selected media | | | | | | | |
|  |  |  |  |  |  |  |  | EP | | Electroporated cells incubated in selected media | | | | | | | |
|  |  |  |  |  |  |  |  | pDNA+EP | | Electrotransfered cells with plasmid DNA incubated | | | | | | | |
|  | | | | | | | |  | | in selected media | | | | | | | |
| Sample | | |  | |  | | |  | |  | | |  | |  | |  |
| Description | | | | | | | | For each experimental and control groups at least 3 RNA | | | | | | | | | |
|  |  |  |  |  |  |  |  | extractions were performed. According to midterm results the | | | | | | | | | |
|  |  |  |  |  |  |  |  | extra semples of RNAs for some groups were extracted. | | | | | | | | | |
|  |  |  |  |  |  |  |  | RT-PCR was performed at least three times for each estimated gene. | | | | | | | | | |
| Nucleic acid extraction | | | | | | | | | | | | | | | | |  |
| Procedure/kit | | | | | | | | RNA wa extracted with TRIzol® Plus RNA Purification Kit | | | | | | | | | |
|  |  |  |  |  |  |  |  | (Thermo Fisher Scientific) | | | | | | | | | |
| Quantification | | | | | | | |  | |  | | |  | |  | |  |
|  |  |  |  |  |  |  |  | Epoch Microplate Spectrophotometer, BioTek Instruments | | | | | | | | | |
| Purity | | | | | | | |  | | | | | | | | | |
|  |  |  |  |  |  |  |  | 260/280 analysis | | | | | | | | | |
| Reverse transcription | | | | | | | | | | | | | | | | |  |
| Procedure/kit | | | | | | | | SuperScript® VILO cDNA Synthesis Kit, Thermo Fisher Scientific | | | | | | | | | |
|  |  |  |  |  |  |  |  |  |  |  |  |  |  |  |  |  |  |
| Amount of RNA | | | | | | | | 500 ng of total mRNA | | | | |  | | | | |
| Reaction volume | | | | | | | | 20 μl | | | | |  |  |  |  |  |
| Temperature and time | | | | | | | | 25°C for 10 minutes | | | | |  |  |  |  |  |
| Storage condition of cDNA | | | | | | | | 42°C for 60 minutes | | | | |  |  |  |  |  |
|  |  |  |  |  |  |  |  | 85°C for 5 minutes | | | | |  |  |  |  |  |
|  |  |  |  |  |  |  |  | -20°C | | | | |  |  |  |  |  |
| Real Time RT-PCR target information | | | | | | | | | | | | | | | | |  |
| Mark | | | Full name | | | | |  | |  | | | References | | | |  |
| TLR9 | | | Toll like receptor 9 | | | | | | | | | | Hemmi et al., 2000 | | | | |
| RIG1 | | | Retinoic acid-inducible gene I | | | | | | | | | | Ablasser et al 2009 | | | | |
| DDX60 | | | DEAD (Asp-Glu-Ala-Asp) Box Polypeptide 60 | | | | | | | | | | Miyashita et al 2011 | | | | |
| DHX9 | | | DExH-Box Helicase 9 | | | | | | | | | | Kim et al 2010 | | | | |
| DHX36 | | | DEAD/H (Asp-Glu-Ala-Asp/His) Box Polypeptide 36 | | | | | | | | | | Kim et al 2010 | | | | |
| AIM2 | | | Absent in melanoma 2 | | | | | | | | | | Burckstummer et al 2009 | | | | |
| cGAS | | | Cyclic GMP-AMP Synthase | | | | | | | | | | Sun et a, 2013 | | | | |
| DAI/ZBP1 | | | DNA-dependent activator of IFN-regulatory factors | | | | | | | | | | Takaoka et al 2007 | | | | |
| DDX41 | | | DEAD (Asp-Glu-Ala-Asp) Box Polypeptide 41 | | | | | | | | | | Zhang et al 2011 | | | | |
| LRRFIP1 | | | LRR Binding FLII Interacting Protein 1 | | | | | | | | | | Yang et al 2010 | | | | |
| p202 | | | Interferon activated gene 202 | | | | | | | | | | Roberts et al 2009 | | | | |
| p204 | | | Interferon activated gene 204 | | | | | | | | | | Unterholzner et al 2010 | | | | |
| SOX2 | | | Sex determining region Y-box 2 | | | | | | | | | | Xia et al 2015 | | | | |
| MRE11 | | | Meiotic recombination 11 homolog | | | | | | | | | | Kondo et al 2013 | | | | |
| Ku70 | | | Lupus Ku autoantigen protein p70 | | | | | | | | | | Zhang et al 2011 | | | | |
| IFNAR1 | | | Interferon Alpha And Beta Receptor Subunit 1 2 | | | | | | | | | | Platanias 2005; Parker et al. 2016 | | | | |
| IFNβ1 | | | Interferon β 1 | | | | | | | | | | Desmet 2012; Parker et al. 2016 | | | | |
| TNFα | | | Tumor necrosis factor α | | | | | | | | | | Wajant et al; 2002 | | | | |
| IL1β | | | Interleukin 1 β | | | | | | | | | | Hornung 2010 | | | | |
| RT-PCR oligonucleotides | | | | | | IDT Oligonucleotides (IDT, Coralville, IA, USA) | | | | | | | | | | | |
| Primer  sequences | | TLR9 | | | | mTLR9-88F | | | | | GAATCCTCCATCTCCCAACAT | | | | | | |
|  |  |  |  |  |  | mTLR9-179R | | | | | CAGAGTCTCAGCCAGCAC | | | | | | |
|  |  | RIG1 | | | | mRIG1-1581F | | | | | GCTGCCATGCAGAGTGATTGGAAA | | | | | | |
|  |  |  |  |  |  | mRIG1-1749R | | | | | ACTCTTGGCCACACAGCTGTAGAA | | | | | | |
|  |  | DDX60 | | | | mDDX60-4160F | | | | | ACTGGAACACTCGCTTTGG | | | | | | |
|  |  |  |  |  |  | mDDX60-4306R | | | | | GAAGTAGACATCACCCAACAGG | | | | | | |
|  |  | DHX9 | | | | mDHX9-for | | | | | AAACTCCCCATTGAACCTCG | | | | | | |
|  |  |  |  |  |  | mDHX9-rev | | | | | TGTATCCCAGGCGTTTTCC | | | | | | |
|  |  | DHX36 | | | | mDHX36-for | | | | | CCCAAAGTTGCTAAAATCCGAC | | | | | | |
|  |  |  |  |  |  | mDHX36-rev | | | | | CCAGTTGTAGTGGAAGTCTGTC | | | | | | |
|  |  | AIM | | | | mAIM2-807F | | | | | CCACCCGCAGTGACAATGACTTTA | | | | | | |
|  |  |  |  |  |  | mAIM2-954R | | | | | GCTTTCAGCACCGTGACAACAAGT | | | | | | |
|  |  | GAS | | | | mcGas-for | | | | | GTGAGGACCAATCTAAGACGAG | | | | | | |
|  |  |  |  |  |  | mcGas-rev | | | | | AGCATGTTTTCTCTATCCCGTG | | | | | | |
|  |  | DAI/ZBP1 | | | | mDAI-1081F | | | | | TGCTTTCTAGAGGACGCCACCATT | | | | | | |
|  |  |  |  |  |  | mDAI-1213R | | | | | TGGCTTCAGAGCTTGTACCTGTGT | | | | | | |
|  |  | DDX41 | | | | mDDX41-for | | | | | AGTGCTCATGGACCTCAAAG | | | | | | |
|  |  |  |  |  |  | mDDX41-rev | | | | | CTCCACAGAAGGCACAGC | | | | | | |
|  |  | LRRFIP1 | | | | mLRR-462F | | | | | CCAGTTTGCCGAAGTGAAAG | | | | | | |
|  |  |  |  |  |  | mLRR-559R | | | | | CTCTCCGTTGGTGGCTATTT | | | | | | |
|  |  | p202 | | | | mp202-1145F | | | | | CCCGGGAAACACCATTGCTTTATCAG | | | | | | |
|  |  |  |  |  |  | mp202-1233R | | | | | TCTTCACCTCAGACACGCTGGAAT | | | | | | |
|  |  | p204 | | | | mp204-693F | | | | | CCAGTCACCAATACTCCACAG | | | | | | |
|  |  |  |  |  |  | mp204-831R | | | | | GAGCACCATCACTGTCAGG | | | | | | |
|  |  | SOX2 | | | | mSOX2F | | | | | CACATGGCCCAGCACTAC | | | | | | |
|  |  |  |  |  |  | mSOX2R | | | | | CCCTCCCAATTCCCTTGTATC | | | | | | |
|  |  | MRE11 | | | | mMRE11F | | | | | CAGAAAGGAAGCACAAAACTCG | | | | | | |
|  |  |  |  |  |  | mMRE11R | | | | | TCACAAACATCCGATAGAGCC | | | | | | |
|  |  | Ku70 | | | | mKu70F | | | | | CGGGAAACAAATGAACCAGTG | | | | | | |
|  |  |  |  |  |  | mKu70R | | | | | TCCTCTTTCTCCAGCACAATC | | | | | | |
|  |  | IFNAR1 | | | | mIFNAR1F | | | | | TCTCTGTCATGGTCCTTTATGC | | | | | | |
|  |  |  |  |  |  | mIFNAR1R | | | | | CTCAGCCGTCAGAAGTACAAG | | | | | | |
|  |  | IL1β | | | | IL1b-140f | | | | | AGTTGACGGACCCCAAAAGA | | | | | | |
|  |  |  |  |  |  | IL1b-232r | | | | | TGCTGCTGCGAGATTTGAAG | | | | | | |
|  |  | IFNβ1 | | | | IFNb1-241F | | | | | TGCCATCCAAGAGATGCTCCAGAA | | | | | | |
|  |  |  |  |  |  | IFNb1-364R | | | | | AGAAACACTGTCTGCTGGTGGAGT | | | | | | |
|  |  | TNFα | | | | TNFαF | | | | | CCCTCCAGAAAAGACACCATG | | | | | | |
|  |  |  |  |  |  | TNFαR | | | | | GTCTGGGCCATAGAACTGATG | | | | | | |
| Real time RT-PCR Protocol | | | | | | |  | |  | | |  | |  | |  | |
|  |  | | |  | | |  | | SYBR Select Master Mix (Thermo Fisher Scientific) | | | | | | | | |
| Reaction volume | | | | | | | | | Reaction volume | | | | | 20 μl | | | |
| Primer concentration | | | | | | | | | Primer: | | |  | | 100 nM | | | |
| Complete thermo cycling parameters | | | | | | | | | Hold: | | | 50°C 2 min | | | | | |
|  |  |  |  |  |  |  |  |  |  |  |  | 95°C 2 min | | | | | |
|  |  |  |  |  |  |  |  |  | 40 cycles: | | | 95°C 15 sec | | | | | |
|  |  |  |  |  |  |  |  |  |  |  |  | 60°C 15 sec | | | | | |
|  |  |  |  |  |  |  |  |  | Extend: | | | 72°C 1 min | | | | | |
| Real time RT-PCR instrument | | | | | | | | | QuantStudio 3 Real-Time PCR system | | | | | | | | |
|  |  |  |  |  |  |  |  |  | (Thermo Fisher Scientific) | | | | | | | | |
| Data analysis | | | |  | | |  | |  | | |  | |  | |  | |
| Analysis of expression | | | | | | | | |  | | | | | | | | |
|  |  |  |  |  |  |  |  |  |  | | | | | | | | |
|  |  |  |  |  |  |  |  |  |  | | | | | | | | |
|  |  |  |  |  |  |  |  |  | \| Relative quantification was performed by the 2-ΔΔCt method [1]. The normalization was performed if Ct value was less than 35. The mRNAs of TLR9, RIG1, AIM (TS/A and WEHI164) and SOX2 (TS/A)  were not detected. \| \| --- \| | | | | | | | | |
| Statistical methods | | | | | | | | | One-way analysis of variance by | | | | | | | | |
|  |  |  |  |  |  |  |  |  | one-way Anova (SigmaPlot 12.0) | | | | | | | | |
